# An Effective Data Science Technique for IoT-Assisted Healthcare Monitoring System with a Rapid Adoption of Cloud Computing

**DOI:** 10.1155/2022/7425846

**Published:** 2022-01-18

**Authors:** Rasha M Abd El-Aziz, Rayan Alanazi, Osama R Shahin, Ahmed Elhadad, Amr Abozeid, Ahmed I Taloba, Riyad Alshalabi

**Affiliations:** ^1^Department of Computer Science, College of Science and Arts in Qurayyat, Jouf University, Sakakah, Saudi Arabia; ^2^Deanship of Research and Graduate Studies, Applied Science University, East Al-Ekir, Bahrain

## Abstract

Patients are required to be observed and treated continually in some emergency situations. However, due to time constraints, visiting the hospital to execute such tasks is challenging. This can be achieved using a remote healthcare monitoring system. The proposed system introduces an effective data science technique for IoT supported healthcare monitoring system with the rapid adoption of cloud computing that enhances the efficiency of data processing and the accessibility of data in the cloud. Many IoT sensors are employed, which collect real healthcare data. These data are retained in the cloud for the processing of data science. In the Healthcare Monitoring-Data Science Technique (HM-DST), initially, an altered data science technique is introduced. This algorithm is known as the Improved Pigeon Optimization (IPO) algorithm, which is employed for grouping the stored data in the cloud, which helps in improving the prediction rate. Next, the optimum feature selection technique for extraction and selection of features is illustrated. A Backtracking Search-Based Deep Neural Network (BS-DNN) is utilized for classifying human healthcare. The proposed system's performance is finally examined with various healthcare datasets of real time and the variations are observed with the available smart healthcare systems for monitoring.

## 1. Introduction

Healthcare monitoring and diagnosis of health became a vital part of the healthcare sector. People do not prefer to visit hospitals because of the time constraint, which may result in many health problems [1]. Early diagnosis and prediction of health are essential to cure many diseases. The healthcare issues are resolved with the presence of a smart healthcare system. Good affordable support of healthcare systems at low cost is expected by the patients in the present smart healthcare system. Such an expectation is satisfied with the innovation of the Internet of Things (IoT), data science, and cloud computing techniques [2]. Data collection and data storage are two eminent problems faced by the healthcare industry. In the screening of the health of the patients, data analytics and data collection play a vital role. Thus, cloud computing and data science techniques are the bases for any healthcare system for overcoming various problems of the healthcare systems, which arise based on technical aspects [3].

**Figure 1:**
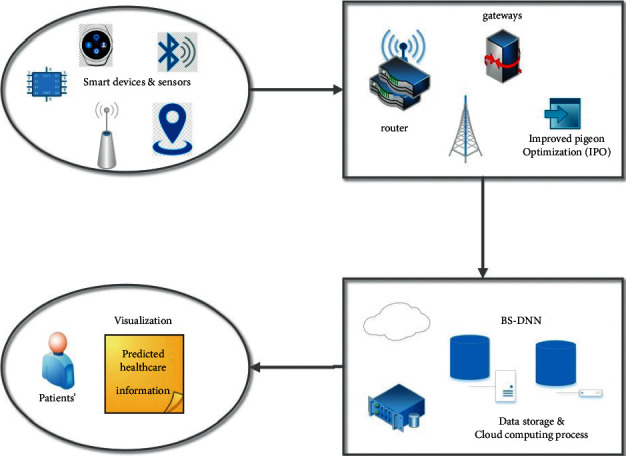
IoT system components.

IoT has many healthcare endeavours, which help the patients, hospitals, physicians, families, insurance companies, and other healthcare professionals. The adherence of the patients could be tracked by the IoT for planning about treatments or any requirement for emerging medical attention [4]. IoT helps the healthcare sector by building a strong relationship between the patients and the healthcare professionals. IoT in modern healthcare involves redesigning with compromising social, economic, and technological aspects [5]. Thus, huge attention has been gathered by IoT in the past years which made striking improvements in the healthcare monitoring system. As the usage of cloud computing grows faster, it is implemented in every field and notably in the healthcare sector. Cloud computing is an economical and efficient technology, which assists in the incorporation of large-scale data and investigating this data for researching genetic tablets [6]. IoT in healthcare utilizes wearable sensors, which gather a large amount of information and the ideal technique for withstanding this huge data is cloud computing. The gathered information is also analyzed with cloud computing as shown in Figure 1.

The development of cloud computing and IoT is enhancing operational efficiency, staff satisfaction, and patient safety [7]. The personalized healthcare monitoring system offers e-health services for fulfilling the assistive and medical requirements of ageing people. IoT is such a striking advancement that assists in various real-time engineering endeavours with improved services. With the usage of IoT, new information could be discovered with an early prediction and this information helps in decision-making for enhancing the quality of life [8]. The eminent objective of this research is to construct an IoT supported healthcare monitoring system with the rapid adoption of cloud computing, which improves data accessibility and efficiency of data processing in the cloud. The initial section of the paper deals with the disquisition review of the studies related to this research [9]. The paper then maps out the IoT supported healthcare monitoring system with the rapid adoption of cloud computing with an effective data science technique [10].

Moreover, the creation and implementation of the healthcare systems via mobile phones are fraught with challenges, such as fantastic information storage and data anomaly, security and protection, administration, access to diverse assets, and interoperability. Cloud computing allows users to access common resources and fundamental infrastructure on an individualized and uncomplicated basis, providing operations in response to requests made via the systems and completing activities in accordance with changing demands. The “Internet of wellness sensor objects” is part of an Internet of things (IoT) based healthcare monitoring architecture. These devices generate massive amounts of information that a physician would be unable to handle. The physician's primary worry is that he must make decisions regarding a patient's healthcare, which necessitates isolating information about one particular patient from a flood of medical information pertaining to a large number of patients. In this case, an IoT controller would be used to transfer medical information to the clouds, which will handle the massive volume of data and make big data analysis possible. These data processing and analytics enable continuous monitoring of the patient's condition.

The remainder of the paper is organized as follows: Section 2 deliberates the recent literatures of healthcare monitoring system; Section 3 explains the proposed HM-DST mechanism and its workflow, as well as the algorithms for effective healthcare monitoring of the patients; Section 4 is dedicated to the result and discussion, which shows the comparison of the performance metrics of the existing methods with the proposed ones; and, finally, Section 5 concludes the paper.

## 2. Literature Review

The authors of [11] presented the detailed fabrication for constructing the intelligent healthcare systems supported by mobile computing and data analytics. The representative intelligent healthcare endeavours for mapping out the mobile computing and data analytics availability for improving the healthcare service performances is discussed in this paper. Results of this research revealed that the development of mobile computing and data analytics proved the efficiency of the healthcare systems with more convenient and intelligent services and applications. Their study lacked details about the advanced techniques like affective computing, deep learning, and cognitive computing for further improvement of user experience and service quality.

The authors of [12] proposed an IoT assisted healthcare platform for ICU patients during the outbreak of COVID-19. Here the healthcare monitoring is presented in critical situations. The system was integrated with unobtrusive and wearable sensors for monitoring the patients having COVID-19 disease. The research was carried out among the COVID-19 patients of Brazil. The drawback of the research was that although their system was effective, it is not suitable for continuously monitoring health. The research paves way for the integration of machine learning algorithms for predicting risky factors and implementing suitable action faster for maximizing the effectiveness of treatment.

The authors of [13] surveyed analyzing the market trends, applications, and components of IoT in the healthcare sector with the aim to learn about the recent advancements in cloud computing and IoT based healthcare endeavours since the year 2015. The applications of wearables, cloud computing, big data, and so forth in the healthcare sector are studied in this paper. This paper also discovers many policies and regulations about e-health and IoT for determining the assistance of cloud in computing and IoT in the healthcare monitoring sector. The paper recommends considering the attacks, vulnerabilities, and threats for preventing the occurring security risks.

The authors of [14] analyzed the factors that affect the IoT supported smart hospital design. The research mapped out the opportunities, existing technologies, challenges, optimization factors, and also the system architecture that occurs with the application of the IoT technology in smart healthcare applications. The results of the study revealed the drawbacks of the smart hospital model and their associated factors for elimination. The research offers a road map for the researchers, system developers, and managers in optimizing the smart hospital system design.

The authors of [15] presented a combination of IoT and cloud architectures for making smart healthcare systems to support real-time endeavours during performing and processing of artificial intelligence (AI) on the data created with wearable sensors. The paper presents a short literature review on the general application of IoT techniques in healthcare systems, initiating from early health monitoring techniques beginning from the description of wearable sensors to the recent trends in smart health. The review concludes that machine learning and artificial intelligence play a vital role in healthcare systems but their applications need cloud services' assistance.

The key contribution of the proposed work is given as follows:

- Initially, an Improved Pigeon Optimization (IPO) algorithm or alternate data science technique is introduced for data compilation and data storage in the cloud for enhancing the speed of forecasting
- Then the optimal features are described for choosing and gathering the required features
- A Backtracking Search-Based Deep Neural Network (BS-DNN) is utilized for classifying the services of human health
- Finally the proposed system's performance is observed in real time in multiple health databases. The results are finally compared with the recent healthcare monitoring systems

## 3. Proposed HM-DST Methodology

The major objective of this proposed research is to utilize the IoT system with the rapid adoption of cloud computing like the electronic health strategy like the details of the patients. The personal information of the patients includes the heart speed abnormalities, blood mass, telephone digits, marital status, language, age, and name. The information is gathered by multiple sensory plans utilized in the body of the patient. For evaluation and safety concern, the blood test impacts are encrypted and stored in the archive. AES (Advanced Encryption Standard) algorithm performs the encryption of patient's data. For healthcare monitoring, data accumulation and study of data play a vital role in screening the health of patients. Thus, cloud computing and data science techniques are highlighting various issues of the healthcare systems technically. Further enhancement of the healthcare systems is achieved with the implementation of efficient data science technique in the IoT supported healthcare monitoring (IoT-HM) system which is supported with cloud computing, which in turn enhances the performance of data processing and accessibility of the data present in the cloud. IoT makes use of various sensors for collecting real data about the patients. The gathered data is then subjected to cloud storage, from where the data is processed.

**Figure 2:**
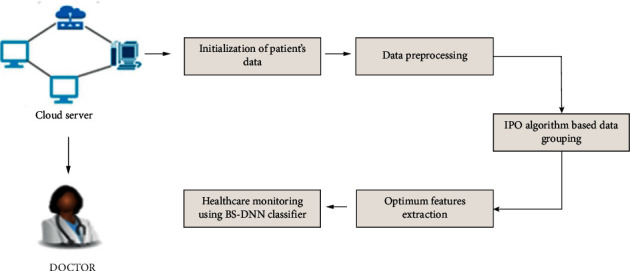
Working process of the proposed HM-DST mechanism.

Figure 2 illustrates the proposed system's architecture. This architecture gives good data with the data science technology with IoT assistance for healthcare monitoring system (HM-DST). Multiple sensors are utilized for monitoring variations in the human body such as heaviness in blood and hotness. This data would be stored utilizing a local method of dispensation, which is regarded as the needed medical data. This is an effective system in the prediction of cancer in patients, such that it could be utilized for creating a mammogram or blood test result after detection of variations in temperature and blood cell. As the blood test features are the features of the cancer patient or a normal person, the features of the results of blood test are classified. On the perception of benefits caused to the users or specific users, this is essential.

The usages of remote sources by the users are determined by the source. The delivery of the computers is done with client-planned agreements like cell phones, tablets, computers, and workstations. A little part of the above tools, which are used by cloud customers, are contemplated on their software or the computer system, which is distributed. In the place of web browser for integration of cloud application, the cloud application with explicit programming of the client is not considered. For the classification purpose, in-depth learning tools like GoogLeNet, VGG16 (a convolutional neural network architecture), and AlexNet are used. The structure of the neural network is designed for identifying various tumours in cancer patients like leukemia, lung cancer, and breast cancer. If there is a cloud storage for storing this essential data, then there is no need for getting details about treatment. This cloud storage has a direct access, which is available at any time where high processing or interruption is not there.

### 3.1. Grouping of Cloud Storage with Improved Pigeon Optimization (IPO)

The Pigeon Inspired Optimization (PIO) algorithm is a newly recommended Swarm Intelligence (SI) algorithm that highlights the incoming features of the pigeon algorithm. Two operators are created utilizing precise rules that encapsulate pigeon features, for example, firstly, a scope worker and a map, which reflect the incoming characteristic of magnetic and Sun particles, and, secondly, a familiar income behaviour as a stage operator. The magnetic field's action enables us to estimate the position or height or direction of the magnetic field. The field on Earth could be understood by the pigeons with the help of a magnet for creating a brain map. On their perception, the variation of direction of the compass indicates the Sun's height. They are contemplated on the Sun and the magnets are at a far distance from their home. The scope worker and the map are designed for reflecting such incoming feature. In the proposed system, the new IPO algorithm and the Particle Swarm Optimization (PSO) algorithm are employed for improving the capabilities of optimization and the search capabilities globally. The learning process for the Improved pigeon Optimization (IPO) algorithm is given below.

The PSO settings are launched and updated and then the adaptive mutation is enabled in the PSO. PSO pieces initiate from the particular probability of every contact. The expansion of the particle search space and diversity of PSO demography are improved with the functionality of adaptive mutation. The status and speed of PSO are updated in the two following equations:

(1) $$\begin{array}{c} P_{r}\left(T+1\right)=P_{r}\left(T\right)\alpha +a_{1}\;{\text{rand}}_{1}\;\left(V_{1}\left(T\right)-Q_{r}\left(T\right)\right)+a_{2}\;{\text{rand}}_{2}\left(V_{\text{gl}}\left(T\right)-Q_{r}\left(T\right)\right). \end{array}$$

(2) $$\begin{array}{c} Q_{r}\left(T+1\right)=P_{r}\left(T\right)+Q_{r}\left(T\right). \end{array}$$

In the above equations, *P*_*r*_(*T*) and *Q*_*r*_(*T*) indicate the speed of the *r*th atom and the location of the *r*th atom in the iteration *T*. The inertia weight is represented as *α*. *a*1 and *a*2 are the speeding factors of the Pigeon Swarm Optimization (PSO) and these values are always higher than 0. *V*_*l*_ indicates the best location of the character and *V*_gl_ indicates the global best place. The two random figures of values 0 and 1 are represented as rand_1_ and rand_2_. The IPO algorithm parameters are initialized and encapsulated under worldwide best morals. IPO is utilized for updating the settings. The variation in weight is encapsulated under the IPO algorithm. The balance between IPO local and global searches is affected by the recession weight. The variable weight formula is given as follows:

(3) $$\begin{array}{c} \alpha \left(T\right)=\alpha_{u}-\left(\alpha_{u}-\alpha_{z}\right)\times \frac{T}{t}. \end{array}$$

The inactivity heaviness is indicated as *α*(*T*) in iteration *T*. The initial inactivity weight is indicated as *α*_*u*_ and the inertia heaviness as *α*_*z*_ during stop of the iterations. The number of iterations is represented by *t*. A landmark operator, a scope operator, and a map are encapsulated under the IPO algorithm. The position and range of the speed of the range worker and the map are updated as in the two following equations:

(4) $$\begin{array}{c} P_{m}\left(T+1\right)=\alpha \left(T\right).P_{m}{\left(T\right)}^{-\text{RT}}+{\text{rand}}_{3}\left(Q_{t}-Q_{u}\left(T\right)\right). \end{array}$$

(5) $$\begin{array}{c} Q_{m}\left(T+1\right)=P_{m}\left(T+1\right)+Q_{u}\left(T\right). \end{array}$$

In the above equations, *Q*_*m*_(*T*) and *P*_*m*_(*T*) indicate the *m*th pigeon's location and speed, respectively. rand_3_ represents accidental number whose values are 0 and 1. The scope and the map factor is *R*. the finest answer is found when the extent operative and the amp touch the highest number *t*1. The landmark operator with the updated splash of the pigeon is indicated in the three following equations:

(6) $$\begin{array}{c} J_{\text{landmark}}\left(T+1\right)+J_{\text{landmark}}\frac{\left(T\right)}{2}. \end{array}$$

(7) $$\begin{array}{c} Q_{\text{pjz}}\left(T\right)=\sum Q_{m}\left(T\right).s\frac{\left(Q_{m}\left(T\right)\right)}{J\Sigma s\left(Q_{m}\left(T\right)\right)}. \end{array}$$

(8) $$\begin{array}{c} Q_{m}\left(T+1\right)={\text{rand}}_{4}.\left(Q_{\text{pjz}}\left(T+1\right)-Q_{m}\left(T\right)+Q_{m}\left(T\right)\right). \end{array}$$

The pigeon quantities are decreased in the landmark worker procedure by every half in each iteration. *J*_landmark_ (*T*) indicates the pigeons' number in iteration *T*. *Q*_pjz_ indicates the pigeon's center position. The fitness purpose is indicated by *s* (.). rand_4_ is the random number having values from 0 to 1. The IPO algorithm ends when the maximum landmark operator (*t*_2_) is reached by the IPO algorithm and this value represents *V*_gl_, which is global optimal solution, which is the optimization end result. The local optimization chance is increased with the use of the Gaussian breach. The added individual with the Gaussian barrier is given in the following equation:

(9) $$\begin{array}{c} Q_{\text{ns}}\left(T\right)=Q_{n}\left(T\right)+\log\text{ Sig}\frac{\left(J_{p\max}/-T+2\right)}{y}.J\left(\beta ,\gamma \right). \end{array}$$

Chance *X*_*c*_ indicates the poorer personality in the simulated annealing replica. It is given in the following equation:

(10) $$\begin{array}{c} X_{c}=\exp\left(\frac{\Delta h}{t}\right) \end{array}$$

In the above equation, Δ*h* is the annealing heat and this reduces with the increase in the rearrangement. The local optimization is exited with an innovative Algorithm 1, which is given below.

### 3.2. Extraction of Feature and Feature Selection

The extraction of the features involves choosing the input picture in the form of a miniature that protects the original image's features. After identification, the process of acquiring the characters is minimized. The input properties, which are highly active, are regarded as attribute selection and attribute extraction, which increases the variables of the class and minimizes the class variance. The most eminent data are eliminated for taxonomic works, minimizing the variability in class structure and then increasing it. The calculation costs and storage space are reduced by smaller space. The multivariate time series is converted into univariate time series.

### 3.3. BS-DNN Based Healthcare Monitoring

The health status of the patients is categorized here and the best guidance is given to the patients with the help of IoT assisted cloud computing. The collected data is then processed with decision-making technique. Thus, Artificial Neural Network is employed for the similar classification. The backtrack search is combined with DNN here for computing the optimal solution in the process. Let us assume the following:

*p*_*x*_^*a*,*b*^=[*p*_*a*,*b*_(0, *x*) … *p*_*a*,*b*_(*B* − 1)*x*]^*t*^ such that *o* ≤ *x* ≤ *n* − 1. Here, *p*_*x*_^*a*,*b*^ denotes the nonzero rudiments. The corresponding passage matrix *Q*_*a*,*b*_ is given as follows:

(11) $$\begin{array}{c} Q_{a,b}\left[\text{Pa},b\left(0,0\right)\cdots \text{Pa},b\left(1,0\right)\vdots \ddots \vdots 0\cdots \text{Pa},b\;\left(0,n-1\right)\right]. \end{array}$$

The background search algorithm encourages the bird with its produced attractive terms and its violent approach. The row indicator is denoted as *M*_*a*,*b*_=[*m*(1) … …*m*(*M*)]. The resultant equation is given as follows:

(12) $$\begin{array}{c} P_{x,\text{pp}}^{a,b}=\left[R\left[m\left(c\right),X\right],R\left[m\left(c\right),X\right]\right]. \end{array}$$

Here the resultant matrix is denoted as *R*, which helps in processing the requests of the users. The optimizations of the constraints are done by DNN. The grouping way, which is utilized for calculating the new summit position, is known as test point and the optimal performance. The new test point is calculated with the following formula:

(13) $$\begin{array}{c} n_{r}=\left\{Tr,\text{if }F\left(\text{Tm}\right)<F\left(\text{nr}\right)\text{nr Otherwise}\right\}. \end{array}$$

Elements present in a specific point or direction help in activating the search engines and offer general access to the package. The new tests, which are chosen for the next process, are contemplated on the following condition:

(14) $$\begin{array}{c} n_{r}=\left\{\text{Tr},\text{if }F\left(\text{Tm}\right)<F\left(\text{nr}\right)\text{ nr otherwise}\right\}. \end{array}$$

After optimization of the time constraint, the won strength of every user is calculated as follows:

(15) $$\begin{array}{c} D_{f}=n_{1}+n_{2}+\Lambda ,\text{where }f=k_{1},k_{2},\ldots k_{n}. \end{array}$$

Finally the outcome of the optimization is indicated as follows:

(16) $$\begin{array}{c} \text{OR}=\max\left[D_{f}^{1},D_{f}^{2},D_{f}^{\beta },\Lambda D_{f}^{x}\right]. \end{array}$$

## 4. Results and Discussion

**Figure 3:**
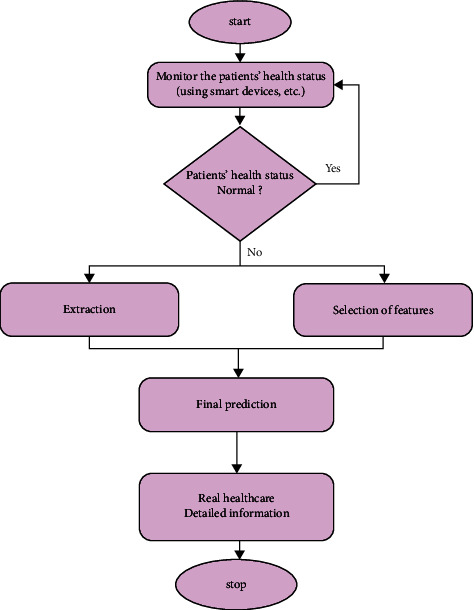
Flow chart of the proposed model.

The performance evaluation of the proposed system is shown in Figure 3 and also the test system is dealt with in this section. The test section is divided into four parts: statistical analysis of warning section, prediction analysis of blood pressure attack, data granulation performance, and data mining. The test section is suitable for cloud endeavours such as built-in, dynamic, and diverse client requirements (QoS). Various applications possess various operating levels, dynamic program scaling needs, and workflows. Thus, many enterprise and management models utilize cloud for storage of host data. Complex connections are created by the cloud and the cloud then transfers and arranges the requirements. Cloud dispensation situations are displayed and reproduced by the Cloud SIM, which is a famous tool. It delivers a social and structural performance of many discovered areas. The information about blood pressure activities and patient symptoms does not exist on the Internet database. This incompatibility paved way for the addition of many databases for evaluating the database structure. This Cloud SIM has three types of databases: the functional database, chronic kidney database, and diabetes database. The patient database is systematically embedded into these three databases, for checking the functioning of a particular system.

- Cloud storage activates the data stored in the cloud folder that could be monitored at any time.
- The predictive model helps in predicting behaviour effectively and architecturally. Thus, health here is regarded as a dynamic factor that is of great importance.
- The real-time perspective offers information on time subject to the present user condition at present.
- IoT sensor is utilized for collecting the information regarding dietary conditions, location, environment, and health of the patients at any time.
- The major contributor offers the necessary information regarding a specific research contribution.
- The application domain provides information regarding a specific domain.

### 4.1. Description of the Dataset

After expansion to the neural network, there are three layers for predicting the blood pressure such as output, night, and input layers. One among nine injections are used for predicting a hypertension attack's severity. Among the nine entry points, eight of them are obesity, severe headache, irregular heartbeat, kidney disease, blood glucose, liver disease, heart illness, and sickness, and the remaining one is the hypertension. The isolation BS-DNN is done in place of high, moderate, or low risk of hypertension. Among the 5000 datasets for the preparation data, 3000 datasets are utilized and the remaining datasets are utilized for testing purpose. The 3000 datasets are trained at the speed of 3.7 with BS-DNN with various repetitions, learning speeds, and motivations. Firstly, the trial is conducted for above 1000 repetitions with various learning speeds and repetition speeds.  Table 1 indicates these values.

The results of testing phase indicate that the total square error reduces by 0.35 and 0.65 learning and repetition speeds, respectively. Secondly, the case is conducted for 2000 repetitions with various repetition and learning speeds. The results indicated that very low error was caused at 0.85 and 0.15 knowledge speeds. The third testing phase results indicated a low quadratic error of 0.85 and 0.15 knowledge speeds. The experiment was repeated for 4000 repetitions as well. Lowest errors were shown in the results with the learning speed value of 0.95 and error of 0.05. Minimum error values were obtained by repeating the tests for over 5000 repetitions and 6000 repetitions. A significant variation was observed with these high repetitions. Thus, 5000 repetitions are recommended to be used with notation rate of 0.85 and study rate of 0.15. After the preparation of the database, examination of 2000 databases was conducted with an audit based on the hypertensive attack risks. BS-DNN is utilized for calculating the hypertension risks with multiple statistical techniques. Classification was properly done in the case defence, root relation square fault, root standard square error, relation absolute mistake, average complete error, kappa statistics, and health records. High *F*-measure, recall, precision, sensitivity, and specificity are offered by BS-DNN, when compared with other models of classification such as linear regression (LR), K-Nearest Neighbour (KNN), Multilayer Perceptron (MLP), and Deep Neural Network (DNN). BS-DNN helps in getting useful characteristics for determining health risks. The categorization accuracy indicates that BS-DNN gives better results when compared with other classification models.

### 4.2. Comparison of Performance

An analysis of warning generation performance was made. The comparative complete error, comparative quadratic mistake, typical root square mistake, average complete mistake, security, accuracy, uniqueness, and compassion are learned by the particular model. The particular model provided a high efficiency with accuracy, coverage, specificity, and sensitivity. Errors are less during warnings like complete mistake, square root mistake, standard mistake, and average complete mistake.  Table 2 shows a comparison of the performances of the existing and proposed classifiers.

**Figure 4:**
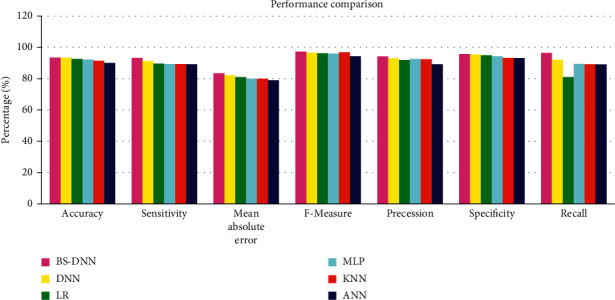
Comparison of the performances of the proposed and existing classifiers.

Figure 4 shows the comparison of results of the proposed and existing classifiers.

The above figure indicates that the sensitivity of BS-DNN is high compared with those of the other classifiers. The specificity of BS-DNN is also high when compared with those of the other classifiers. Similarly, the recall and scale of BS-DNN classifier are also high when compared with those of the other classifiers.

## 5. Conclusion

This research offered an effective data science technique incorporating IoT and cloud computing for monitoring the health of the patients at any time. Research on IoT assisted healthcare monitoring systems is essential as they are employed in cloud computing which enhances data size while utilizing data storage in the cloud. The proposed system is contemplated on an effective data science technique for IoT assisted healthcare monitoring systems with the rapid adoption of cloud computing. The proposed system helps in increasing the data processing efficiency and also helps in monitoring the health of patients at any time. IoT sensors are fixed on the human body for gathering the needed medical data and this gathered information is stored in the cloud. Initially, an enhanced IPO optimization algorithm is constructed and a PAV optimization algorithm is employed for the clustering of cloud data, which could enhance the speed of forecasting. The optimal sampling and extraction methods for feature extraction and feature selection have been demonstrated. The BS-DNN classification is employed for classifying human health. Better results were obtained from the proposed system when compared with the other available healthcare monitoring systems. After the data collection in the long term, the factors associated with the prediction of potential risks must be explored further for expanding the human healthcare monitoring system application, which is contemplated on IoT. This results in an effective and scientific base for restricting and preventing high-risk diseases in the future.

## Figures and Tables

**Algorithm 1:**
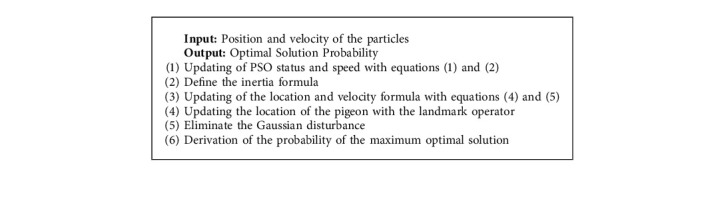
Grouping of cloud data with IPO.

**Table 1:** Total square root error for various iterations.

Momentum	Learning rate	Total square error					
		1000 iterations	2000 iterations	3000 iterations	4000 iterations	5000 iterations	6000 iterations
0.05	0.95	5.862	4.562	5.013	3.124	1.916	1.714
0.15	0.85	5.825	4.213	3.119	4.814	1.016	1.014
0.25	0.75	5.482	5.702	4.216	4.542	2.819	1.891
0.35	0.65	5.989	5.271	4.582	4.819	2.904	2.919
0.45	0.55	5.922	5.535	4.846	4.932	2.819	3.861
0.55	0.45	6.253	5.583	5.056	4.829	2.881	3.901
0.65	0.35	5.216	5.288	5.622	4.916	3.908	2.808
0.75	0.25	6.249	5.313	5.332	5.210	4.560	4.571
0.85	0.15	7.186	6.123	6.115	5.617	4.619	5.851
0.95	0.05	8.214	7.284	7.518	6.181	5.189	5.957

**Table 2:** Comparison of the results of the proposed and existing classifiers.

Metrics in %	BS-DNN	DNN	LR	MLP	KNN	ANN
Accuracy	93.48	93.5	92.6	92.2	91.53	90.13
Sensitivity	93.26	91.22	89.73	89.35	89.28	89.22
Mean absolute error	83.53	82.16	81.06	79.99	79.96	79.06
*F*-measure	97.32	96.52	96.32	96.10	96.90	94.41
Precession	94.21	92.91	91.9	92.6	92.4	89.26
Specificity	95.71	95.36	95.03	94.41	93.25	93.22
Recall	96.41	92.16	80.99	89.41	89.26	89.13

## Data Availability

The data used to support the findings of this study are included within the article.
